# Supplementary material for: The Effects of Ventilation, Humidity, and Temperature on Bacterial Growth and Bacterial Genera Distribution
Source: Int J Environ Res Public Health. 2022 Nov 20;19(22):15345. doi: 10.3390/ijerph192215345 (PMC9691097; doi:10.3390/ijerph192215345)
Supplement: Supplementary file 1 [file ijerph-19-15345-s001.zip › Supplemental Material.pdf]

Figure S1. Cabinet dimension (A) The front view dimension of the cabinet experiment box; (B) The ventilation holes panel dimension of the cabinet experiment box; (C) The up view dimension of the cabinet experiment box; (D) The side view dimension of the cabinet experiment box. The unit is mm.
