# Supplementary figures and images for: The Effects of Ventilation, Humidity, and Temperature on Bacterial Growth and Bacterial Genera Distribution
Source: Int J Environ Res Public Health. 2022 Nov 20;19(22):15345. doi: 10.3390/ijerph192215345 (PMC9691097; doi:10.3390/ijerph192215345)

**A**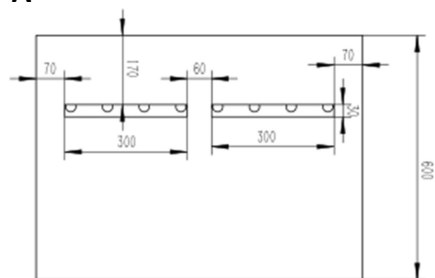**B**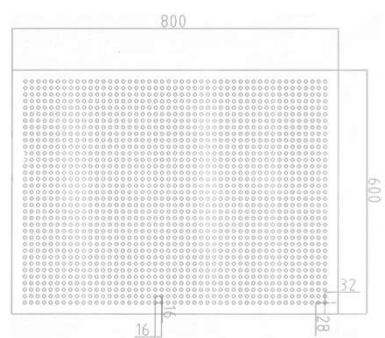**C**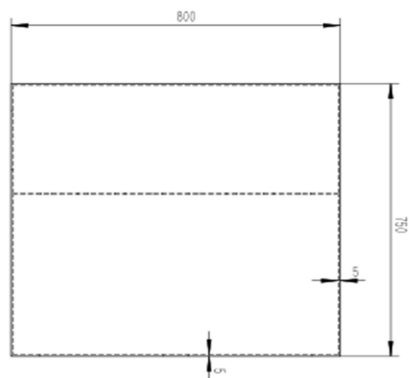**D**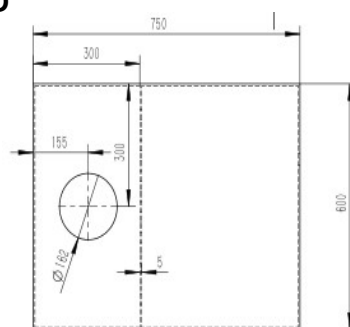

Supplement: Supplementary file 1 [file ijerph-19-15345-s001.zip › Figure S1.pdf]
